# A practical approach to dietary interventions for nondialysis-dependent CKD patients: the experience of a reference nephrology center in Brazil

**DOI:** 10.1186/s12882-016-0282-y

**Published:** 2016-07-16

**Authors:** Lilian Cuppari, Fabiana Baggio Nerbass, Carla Maria Avesani, Maria Ayako Kamimura

**Affiliations:** Division of Nephrology and Nutrition Program, Federal University of São Paulo, and Oswaldo Ramos Foundation, Rua Pedro de Toledo, 282, São Paulo, 04039-000 Brazil; Division of Nephrology, Nutrition Department, Pro-rim Foundation, Joinville, Brazil; Nutrition Institute, Rio de Janeiro State University, Rio de Janeiro, Brazil; Nutrition Program and Division of Nephrology, Federal University of São Paulo, São Paulo, Brazil

**Keywords:** Chronic kidney disease, Diet, Brazil, Dietary protein

## Abstract

This paper describes the 30-year experience on nutritional management of non-dialysis dependent chronic kidney disease (CKD) patients in a public outpatient clinic located in the city of São Paulo, Brazil. A team of specialized dietitians in renal nutrition is responsible to provide individual dietary counseling for patients on stages 3 to 5 of CKD. Two different types of nutrition care protocols are employed depending on the level of renal function. For patients with CKD stage 3 a simplified nutritional assessment is performed and the main dietary focus is on the control of protein intake particularly from animal sources. A more complete nutritional assessment as well as a detailed dietary plan focusing not only on the control of protein but also on energy supply and on specific micronutrients is provided for patients on stages 4 or 5 of CKD. Practical approaches and tools used by the dietitians in our clinic for improving patient´s adherence to protein, sodium and potassium restriction while maintaining a healthy diet are described in detail in the sections of the article.

## Introduction

According to the Kidney Disease Improving Global Outcomes (KDIGO) on evaluation and management of chronic kidney disease (CKD), nondialysis-dependent patients with CKD should be referred to a comprehensive “Conservative Management Program”. This program should embrace multiple actions aiming at slowing/arresting CKD progression; (i) evaluation and management of comorbid conditions; (ii) prevention and management of CKD; (iii) identification, prevention, and management of CKD specific complications (e.g., malnutrition, anemia, bone disease, acidosis); and (iv) planning and preparation for renal replacement therapy (e.g., choice of modality, access-placement and care, preemptive transplantation) [1]. There is convincing evidence that the treatment before the beginning of the dialysis therapy plays essential role toward maintaining better prognosis after the initiation of dialysis or kidney transplant [2]. To this end, a group of health care professional should be engaged and working in an interdisciplinary team. Ideally, the conservative management should include nephrologist, nurse, dietitian, psychologist and physiotherapist or physical educator. This paper will focus on the strategies applied by dietitians that work within an interdisciplinary team in an outpatient clinic situated in São Paulo (Oswaldo Ramos Foundation, Hospital of Kidney, Federal University of São Paulo), Brazil.

In order to understand the nutritional strategies applied to reach the aim of the conservative management program, it is important to give a broad view of cultural and socioeconomic aspects of the patients that attend the outpatient clinic. São Paulo is the largest city of Brazil with close to 12 million inhabitants as estimated in the year of 2015. If one considers the metropolitan area (including the 38 surrounding cities), the number grows up to approximately 19 million of inhabitants. São Paulo attracts Brazilians from all over the country for business, commercial and academic purposes. Therefore, dietitians dealing with dietary issues in the city of São Paulo have to deal with a great diversity of food habits.

Brazil is geographically and culturally divided in 5 regions: North, Northeast, Central West, South and Southeast. Of note, each region has a different availability of vegetables, fruits, grains and so on, which is subjected to the local environmental conditions. Moreover, a diverse immigration in each region was observed during its colonization, which plays a direct influence on food habits. For instance, the North region has big influence on food habits from the South American countries in the surrounding borders, the Northeast which is all surrounded by the sea has a higher consumption of fish and seafood with influence from the Portuguese immigrants and African slaves, while in the Central West the food habit is mainly related to red meat consumption since its main economy in grounded in farming. In the Southeast region, food habits are influenced by the Portuguese, Italian, Japanese and Spanish immigration and the South region by Germany and other Slavic countries.

According to the Brazilian Household Budget Survey (2008–2009) [3], the food items with highest contribution in the purchase of the Brazilian families were, in the following order, beans (a specific type of bean consumed in Brazil), rice, beef, juices (including the non-fresh ones), soft drinks and coffee. Rice and beans are purchased for more than 50 % of the Brazilian families all over the country and are considered the foundation for the main meals (lunch and dinner). Beef is the most consumed animal protein by Brazilians accounting for 70 to 90 % of the population. Green leaves and vegetables represent 10 to 25 % of all food items purchased by families. Temporal trends in food consumption obtained from Household Budget Surveys conducted in Brazil in 1987–1988, 1995–1996, 2002–2003 and 2008–2009, according to purchased food items divided into the extent and purpose of their industrial processing, were classified as: “in natura” or minimally processed foods, processed culinary ingredients, and ready-to-consume processed and ultra-processed food and drink products [4]. Similar to what has been seen worldwide, food acquisition in Brazilian houses has shifted to a lower contribution coming from “in natura” or minimally processed foods, and to a higher contribution coming from ready-to-consume, processed and ultra-processed foods. This was a trend observed in all income quintiles. Therefore, nowadays dietitians working with nondialysis-dependent CKD patients in our outpatient clinic face an even higher challenge since besides planning diets with low content of protein, sodium, potassium and phosphorus there is a need of dealing with the increasing consumption of the ready-to-consume foods which implies in a low quality diet. To that end, nutritional education strategies are constantly developed in order to increase the awareness of adhering to the dietary plan, to make the information more easily understood by the patients and to increase flavor of meals allowing a dietary plan that will not steal the pleasure of eating.

### Characteristics of the CKD outpatient clinic

Our outpatient clinic for treatment of nondialysis-dependent CKD patients is located in the city of São Paulo at the Federal University of São Paulo and Oswaldo Ramos Foundation. Currently, the number of active patients is about 1500 and approximately 90 patients are seen per week by the nephrologists. Those patients are enrolled in the Brazilian public medical care and most of them are low income and low level of education. The team of physicians consists of interns and residents in clinics, graduate students in nephrology and senior nephrologists. The team of dietitians consists of three junior dietitians who are part of the annual training program in CKD and three senior dietitians specialized in kidney diseases that are also involved in research in this field. The decision for referring patients to the dietitians is made by the nephrologists depending on the patient’s need for nutrition intervention; usually those with glomerular filtration rate (GFR) lower than 60 mL/min/1.73 m^2^ are referred. There are no specific criteria for referral but the main reasons are obesity, weight loss and protein energy wasting, poorly controlled diabetes, decreased or progressive renal function, and hyperpotassemia. The first visit with the dietitian is scheduled for a complete nutritional assessment, dietary planning and counseling. Subsequently, patients are monitored routinely in the same day scheduled with the nephrologist in the clinic. Approximately 20 patients are seen per month by the dietitians in the first visit and on average 80 patients are seen per month in the follow-up visits. Besides the nondialysis-dependent chronic kidney disease (NDD-CKD) patients the team of dietitians is also responsible for the nutrition care of patients undergoing hemodialysis (~250 patients) and peritoneal dialysis (~130 patients).

### Protocol of nutrition care

The nutrition care protocol employed in the outpatient NDD-CKD clinic is divided in two levels according to the stage of CKD.

### Level 1

For patients with estimated glomerular filtration rate (eGFR) greater than 30 mL/min/1.73 m^2^ (CKD stage 3) the nutritional assessment includes body mass index (BMI), waist circumference, handgrip strength and the 7-point subjective global assessment. A diet history that includes usual food intake, food habits, clinical and social aspects is obtained through a standardized questionnaire. Laboratory parameters routinely available are serum urea, creatinine, sodium, potassium, calcium, phosphorus, parathyroid hormone, total alkaline phosphatase, hemoglobin, iron, transferrin, ferritin, pH and bicarbonate, lipid profile, glucose, A1c hemoglobin, 25(OH) vitamin D, and protein equivalent of nitrogen appearance (PNA) when 24 h urinary urea is available. At this level of GFR the focus of the dietary counseling is on controlling sodium and protein intake, particularly in regards to protein from animal sources. Therefore, approximately 0.6 to 0.8 g/kg/day with at least 50 % of protein with high biological value (HBV) is calculated. For the HBV foods, lists of protein equivalent items are provided to each patient. In addition, based on the nutritional condition and on the laboratory parameters patients are also instructed on other dietary aspects such as energy, potassium, sodium, sugar, meal planning, healthy quality diet, etc. (details are described ahead). After the first visit the patient is seen by the dietitian in every visit to the nephrologist. In the follow-up visits body weight is measured, adherence to the proposed diet is assessed and adjustments are performed accordingly. Patients are stimulated to actively participate in the decisions. A complete nutritional assessment is performed yearly or when needed. The interval between each visit to the outpatient clinic varies according to the patient´s clinical condition and is usually set by the nephrologist. If the dietitian needs to see the patients in a shorter interval a new appointment is schedule separately. The duration of the first visit and the follow-up visits is on average 60 min and 30 min, respectively.

### Level 2

For patients with eGFR below 30 mL/min/1.73 m^2^ (CKD stages 4 and 5), the nutritional assessment includes the same parameters of Level 1 with the addition of midarm circumference, triceps skinfold thickness and midarm muscle circumference. This subgroup of patients gets a more detailed nutritional assessment due to its higher risk for malnutrition than those with higher GFR. In addition, the appointments with the detailed nutritional assessment are longer and the current number of dietitians is not enough to provide a complete assessment to all patients with GFR > 30 mL/min/1.73 m^2^. Dietary history includes a 3-day food record used to evaluate diet quality, to identify food habits and to calculate particularly protein and energy intake. Clinical and social aspects are obtained through a standardized questionnaire. Laboratory parameters are the same mentioned in the previous item. All data are obtained in the first visit with the dietitian and are essential to prepare a dietary plan. A second visit is then scheduled to provide the patient an individualized and detailed dietary plan with exchange food choices (examples on Tables 3 and 4) and specific recommendations as needed. The prescription of protein and energy are in general of 0.6 to 0.8 g/kg/day and 30 to 35 kcal/kg/day, respectively. Those numbers are used as a guide to calculate the dietary plan. However, if necessary, gradual changes are made during the follow-up visits in agreement with the patient until achieving as close as possible the dietary recommendation with satisfactory laboratory profile and nutritional condition. Each visit takes approximately 30 to 40 min. The interval between each visit to the outpatient clinic follows the same pattern described for patients with CKD on stage 3.

Table 1 summarizes the parameters employed in the nutrition care protocol according to stages of CKD.

**Table 1:** Protocol of nutrition care

CKDstage/Level		3a and 3b Level 1		4 and 5 Level 2	
		Yes	No	Yes	No
Parameters of nutritional assessment	- Body weight and height	*		*	
	- Body mass index	*		*	
	- Waist circumference	*		*	
	- Handgrip strength	*		*	
	- 7-point subjective global assessment (SGA)	*		*	
	- Diet history	*		*	
	- Midarm circumference		*	*	
	- Triceps skinfold thickness		*	*	
	- Midarm muscle circumference		*	*	
	- Diet history and 3-day food records		*	*	
	- Protein equivalent of nitrogen appearance		*	*	
Laboratory parameters	- Serum urea and creatinine	*		*	
	- Sodium and potassium	*		*	
	- Calcium, phosphorus, parathyroid hormone, total alkaline phosphatase and 25(OH) vitamin D	*		*	
	- Hemoglobin, iron, transferrin and ferritin	*		*	
	- Bicarbonate and pH	*		*	
	- Lipid profile	*		*	
	- Fasting glucose and HbA1C	*		*	
	- 24 h urinary urea (when requested)		*	*	
Dietary approaches	- Focus on controlling protein intake by providing lists of protein equivalent food choices.	*			*
	- Individualized dietary plan.		*	*	
	- Lists of protein and energy equivalent food choices.		*	*	
	- Lists of foods with low and high potassium content.	*		*	
	- Tips for reducing sodium intake.	*		*	
Follow-up	Same day of the nephrologist visit or on a separate day depending on nutritional condition.	*		*	

Besides the individualized counseling, patients are encouraged to participate in a group meeting activity scheduled once a month where the dietitian gives a 30-min talk with information about the disease and the importance of the dietary aspects as part of the treatment. This strategy was shown to successfully increase adherence to the low protein diet in the conservative management program in an outpatient clinic in Rio de Janeiro (Brazil) [5].

## Dietary management of NDD-CKD patients

### Management of protein intake

Management of protein intake has been one of the important therapeutic approaches in patients with CKD. Particularly among those under conservative management, the reduction of protein intake has shown to relief the manifestations of uremic syndrome [6]. In addition, clinical trials and meta-analyses have demonstrated that the low protein diet delayed the progression of kidney disease and the initiation of dialysis and was associated with lower risk of mortality [7–9]. Although protein restriction for CKD patients is still a matter of debate among some experts and researchers, claiming at potential risk of developing protein-energy wasting, the risk is minimal when well implemented and tracked regularly by experienced renal dietitians. In addition, there are convincing data showing further indirect benefits such as on proteinuria and metabolic disturbances in patients under a low protein diet [10, 11]. Yet, protein restriction and the low consumption of dairy products and processed foods that contain phosphorus additives by our patients contributes for controlling the amount of phosphorus ingested which is usually less than 700 mg/day.

In line with the most guidelines [12, 13], the amount of protein varying from 0.6 to 0.8 g/kg/day has been recommended in our service for patients with CKD stages 3 to 5 (not dialysis). For those in the early stages of the disease (stages 1 and 2) a diet with 0.8 to 1.0 g/kg/day of protein, as suggested for health individuals, has been recommended. Particularly for those patients with inadequate control of diabetes or any catabolic condition, the amount of protein offered is around 0.8 g/kg/day or a few grams higher in order to offset the increase in muscle catabolism. Of note, for any patient whose loss of urinary protein is greater than 3 g/day, 1 g of protein is added in the diet for each 1 g protein eliminated in the urine. In this context, it is important that the 24-h proteinuria is reassessed during the follow-up to guarantee adequate adjustments. And finally, regardless of the amount of overall protein in the diet, at least 50 % of them should be based on animal origin foods (high biological value proteins) [14].

An alternative dietary approach for patients with CKD, especially for those with glomerular filtration rate below 30 ml/min, is the very low protein diet, containing 0.3 g/kg/day (most vegetal protein) supplemented with a mixture of ketoacids and essential amino acids. The advantage of this therapeutic against the conventional low protein diet is the more prominent reduction of uremic compounds and consequently of uremic symptoms, in addition to improvement of insulin sensitivity, metabolic acidosis and bone and mineral disorders [14].

More recently, reduction of morbidity related to emergency access to dialysis with very low protein diet supplemented with ketoacids has also been demonstrated [15]. We had a positive experience in a protocol conducted in our service by showing significant reduction in serum urea and urinary phosphate with the maintenance of nutritional parameters during 4 months under ketoacids therapy [16]. However, the large number of pills (1 tablet per 5 kg body weight), high cost and dietary monotony make it cumbersome in the routine care of CKD patients.

Poor adherence to the low protein diet is a common feature among our patients as in the rest of the world. Particularly in Brazil, the traditional almost daily-based consumption of beans (Brazilian beans), beef and poultry contributes to the scenario. On the other hand, milk and dairy products consumption is not elevated in Brazil. The former is related to food habit (milk consumption has been typically very low in the general population) and the later (e.g., cheese) is due to the cost since our patients have in general low income. Either way, based on patient’s preference, milk and dairy products are advised exceptionally to alternatively replace meat along the therapy and patients are continuously tracked, particularly in respect to serum phosphorus and PTH. Finally, protein management is somehow a challenge and, in the same time, the cornerstone for the diet planning for CKD patients. An example of a diet with qualitative emphasis for NDD-CKD patients is provided in Table 2.

**Table 2:** Example of qualitative diet for a CKD patient, with quantitative focus on protein

Breakfast	
Bread	
Butter or margarine^a^	
Milk (~70 ml) with coffee	
Snack	
Toast or cracker	
Tea/coffee	
Lunch	
Rice	
Bean	
Raw salad	
Cooked vegetables	
Beef/chicken/fish (~45 g)	
Fruit	
Snack	
Toast or cracker	
Tea/coffee	
Fruit	
Dinner	
Rice or pasta	
Raw salad	
Cooked vegetables	
Beef/chicken/fish (~45 g) or egg	
Fruit or any dessert free from milk or egg	

^a^To be used in small amounts and preferably margarine brands made with healthy vegetable oils

Although we initially prescribe protein around 0.6–0.8 g/kg/day as a guide, when the patient´s usual intake is high, a gradual decrease is prioritized in order to reach the target. Patients are also advised to eat the total serving of protein at once in a meal, if preferable. We emphasize the importance of keeping constant the total daily consumption of the recommended protein intake in order to improve the adherence. Of note, non-animal source of protein is not closely monitored, except beans due to the great content of potassium, since it is a Brazilian habit to eat beans with the broth it was boiled. Lastly, the dietary plan is individualized and lists of substitutes for the main protein servings are provided for our CKD patients, as exemplified in Table 3.

**Table 3:** List of substitutes for half portion (45 g) of medium steak

Food item	Amount (g)	Household measure
Chicken fillet	42	½ medium fillet
Chicken (skinless)	41	1 small thigh
	48	2 wings
	56	1 small drumstick
Cooked beef	56	2 medium cubes
Ground beef	42	3 soup spoons
Meatball roast	50	1 medium unit
Meat dumpling	90	3 small units
Home-made hamburger	40	1 medium unit
Lean piece of pork	50	½ chop
Sirloin roast	45	1 medium slice
Grilled liver	50	½ large steak
Boiled egg	100	2 units
Grilled fish	42	½ medium fillet
^a^Tuna in water	40	4 soup spoons
^a^Sausage	70	2 units
^a^Ham	40	2 thin slices
^a^Hard cheese	60	4 slices
	60	2 medium slices
Ice cream	100	2 balls
Bean	308	2 medium shells
Chickpeas / pea	150	1 medium shell
Soy	76	2 serving spoons
Tofu	110	2 thick slices

^a^These foods should be avoided due to the high content of salt and/or fat

### Management of energy intake

If in one hand adverse clinical conditions such as inflammation, diabetes and hyperparathyroidism are shown to increase the resting energy expenditure (REE) of CKD patients [17–19], factors such as reduction of renal tissue, changes in muscle metabolism due to uremia and sedentary lifestyle counteract by reducing energy expenditure [20, 21]. Having that in mind, the energy recommendation for CKD patients has been maintained as 30 to 35 kcal/kg/day, as indicated for healthy individuals with light physical activity level. In fact, it has been recommended that the daily energy intake should be around 30 kcal/kg/day for those aged ≥60y and around 35 kcal/kg/day for those aged <60y [12]. This approach has been adopted in our service in accordance with the most practical guidelines for CKD patients, but also supported by our established line of research on energy metabolism in the past years based on our Brazilian CKD groups of patients [22–28].

Ideally, to estimate the energy requirement of the patient with CKD factors such as age, sex, nutritional status, level of physical activity, metabolic disorders and comorbidities should be considered. However, the exact contribution of those elements to the total energy expenditure is unknown for CKD population. Therefore, prediction equations for estimating REE could be an alternative for the estimation of energy needs of patients with CKD. We previously compared the most used equations of REE [(i) proposed by Harris and Benedict, and (ii) proposed by Schofield that is currently recommended by the World Health Organization (WHO) in 281 CKD patients (124 not on dialysis, 99 hemodialysis and 58 peritoneal dialysis), and found that those equations overestimated the measures in comparison with indirect calorimetry [29]. Nonetheless, there is still a need of developing population-specific equations in order to adequately estimate the energy requirement of CKD patients. As proposed by the European Best Practice Guideline on Nutrition [13], if one wants to individualize the estimation of energy requirements of CKD patients it is also possible to use the factor for physical activity level (PAL) as proposed by WHO (sedentary or light activity lifestyle PAL: 1.40–1.69). We previously demonstrated in studies including Brazilian patients on hemodialysis a value of PAL in the lower limit of the threshold proposed by WHO [30, 31]. However, this requires further investigation in NDD-CKD patients.

As stated above, the proposed values of 30 to 35 kcal/kg/day has been used for guiding the initial dietary planning for CKD our patients. Nevertheless, the optimum way to evaluate the estimated energy requirement is to monitor the nutritional status of the patient and perform adjustments as required. Of concern, the issue of energy intake becomes especially important when dietary protein restriction is prescribed. Thus, particularly for nondialyzed stages of CKD, where patients are commonly subjected to control of protein intake, it is crucial to ensure provision of enough amount of energy in order to avoid negative nitrogen balance. Even for patients aiming at losing weight, diets with less than 25 kcal/kg/day are avoided. For overweight/obese patients when necessary, we advise a small and gradual loss of body weight in conjunction with physical exercise in order to avoid loss of lean body mass.

Finally, as part of the individual dietary management, lists of food substitutes for the main sources of carbohydrates are also provided for our CKD patients. And, when energy requirement is not reached, foods with great amount of energy and low amount of protein are encouraged (Table 4) as appropriate, and oral energy supplements such as maltodextrin has been used. It is important to mention that besides protein and energy dietary managements, reinforcing general diet quality (including preferences for wholesome foods) and stimulation of physical activity, by the support of the health care team, have been a continuously process.

**Table 4:** Foods with high energy content and low amount of protein

	Amount (g)	Household measure	Energy (kcal)	Protein (g)
Cooked cassava	130	3 medium pieces	154	1.2
Cassava flour	40	2 table spoons	142	0.7
Vegetable oils	8	1 table spoon	71	0
Margarine/butter^a^	5	1 tea spoon	36	0
Table cream (17 to 20 % fat)^a^	20	1 table spoon	50	0.5
Sugar^b^	10	1 tea spoon	39	0
Honey^b^	14	1 tea spoon	43	0
Marmelade^b^	30	1 medium slice	82	0

^a^These foods should be avoided by patients with hyperlipidemia

^b^These foods should be avoided by patients with diabetes and/or hypertriglyceridemia

### Assessment of protein and energy intake

Periodic monitoring of protein and energy intake of patients undergoing dietary control is essential to evaluate the adherence to treatment, the quality of the protein ingested, the quality of the overall diet and to allow adjustments when needed in order to assure long-term successful therapy. The protein and energy intake as well as the quality of the overall diet can be estimated by methods of food intake as the 24-h recall, food surveys, etc. In our clinic, the food frequency questionnaire comprising the main protein sources and the typical daily overall food consumption of the patient are assessed in all visits. The 3-day food diary is applied once a year, together with a more complete nutritional status evaluation including anthropometric measures, subject global assessment and handgrip strength. Specifically for protein intake, a biomarker has been additionally used when the 24 h urine collection is available. Based on the nitrogen urinary excretion, protein equivalent of nitrogen appearance (PNA) is calculated according to the equations as summed up in Table 5.

**Table 5:** Equations to estimate protein equivalent of nitrogen appearance (PNA)

Equation 1 [48]:	
PNA (g protein/day) = 9.35 × G + 11.04	
Where:	
G = Urea nitrogen generation x [urinary urea (g/L)/2.14 x urinary volume (mL) 24 h]/urine collection duration (min) (usually 1440)	
or	
Equation 2 [49]:	
PNA (g protein/day) = [(NUU (g)) + (0.031 g N/kg)] × 6.25	
Where:	
NUU = urinary urea nitrogen x [urinary urea (g/L)/2.14] x urinary volume 24 h (L)	
PNA final values are normalized by ideal or adjusted body weight*	

*Adjusted body weight should be calculated when actual body weight is lower than 95 % adequacy or higher than 115 % adequacy [12]

It is important to emphasize that the protein intake estimated by the PNA must be interpreted with caution, since some conditions as listed below can compromise the results and therefore may not reflect the actual protein intake:

- Incorrect collecting of 24-h urine
- Catabolic conditions (e.g., infection, inflammation, marked weight loss, etc.) overestimate protein intake
- Anabolic conditions (e.g., recovery of nutritional status) underestimate protein intake
- PNA is determined from 1-day urine collection and may not reflect the usual protein intake

### Management of sodium intake

It is well known that control of blood pressure (BP) and proteinuria are pivotal for the preservation of renal function and prevention of complications associated with CKD [32]. Recent randomized controlled trials showed that dietary sodium restriction significantly decreased BP [33, 34] and consistent reductions in proteinuria were also observed in those patients, independent of BP changes [34]. Furthermore, avoidance of excessive dietary sodium intake has been shown to enhance the effect of single-agent renin angiotensin aldosterone system (RAAS) blockade to improve renal and cardiovascular outcomes in two post-hoc analyses of clinical trials [35, 36]. Thus, the assessment and appropriate recommendation of sodium intake are very important approaches to improve outcomes in CKD patients.

### Sodium intake assessment

There are several methods available to measure sodium intake, and the relative strengths and limitations of each method must be taken into consideration before selecting the most appropriate method in a particular context [37]. Dietary assessment methods are easy to perform, but are susceptible to errors related to memory lapses and/or reporting bias [38]. In addition, accuracy also depends on the skills of the interviewer [39]. Repeated 24-h urine measurements are considered to be the gold standard by the WHO, due to their accuracy in quantifying actual intake when correctly collected [40]. However, limitations include high participant burden; high cost of analysis; susceptibility to under or over collection of urine that can introduce error; and inability to account for daily variation in sodium intake if only a single measurement is performed [37].

Since 24-h urine is not often available in clinical practice, dietary assessment constitutes the method with the highest applicability, allowing the investigation of the population dietary’s pattern, including the assessment of regional differences of sodium intake.

In Brazil, the average per capita estimated sodium intake, based on Brazilian Household Budget Survey, did not vary from 2002/2003 to 2008/2009 and reached around 4700 mg of sodium (or 12 g of salt) per day. In both periods, most of the sodium intake was derived from cooking salt or salt-based condiments. Sodium intake from processed foods increased with household purchasing power (9.7 % of total sodium intake in the lower quintile of the per capita income distribution and 25.0 % in the upper quintile). Also, from 2002/2003 to 2008/2009 the relative participation of cooking salt and salt-based condiments in sodium intake was slightly reduced from 76.2 to 74.4 %, while the participation of processed foods increased almost 20 % (15.8 to 18.9 %) [41]. Based on these nationwide data, although differences according to per capita income were observed, at least three quarters of sodium intake was from cooking salt. Therefore, this source of sodium should be evaluated in detail. It is easier to assess when patients cook and/or have meals usually at home. The following are helpful questions to evaluate salt intake at home:

1. One kilogram pack of salt lasts how many weeks or months in your home?
2. How many people usually have most of their meals at home?

Based on these two questions, it is possible to estimate daily individual salt intake. For example: if 1 kg of salt lasts 1 month and this is shared by 4 people, it means that around 250 g of salt is consumed monthly by each one, that corresponds to a daily intake of 8.3 g.

However, if most of meals are out of home, which is very common in larger cities, this method is useless and it is possible to presume that sodium intake is high. Besides, since one quarter of sodium intake comes from processed food, patients might be argued about frequency of intake and portion size of the most common processed food items consumed. As Brazil is a huge country and food habits can vary enormously according to region, it is crucial that the dietitian investigates the most important sodium sources by region.

### Sodium intake recommendation

Most current guidelines, for both general population and specific populations such as those with CKD, recommend a daily sodium intake of <2.3 g/day [42–45] that corresponds to <100 mmol of sodium, <6 g of salt. It means that sodium intake must be reduced if considered the usual intake by the Brazilian population. The following strategies are used in our clinical practice to improve the adherence to low sodium intake.

#### Educational activities

- Although natural spices are easily available in most markets, Brazilians are not accustomed to use them while cooking. Except for garlic, onion and parsley, other spices and herbs are not used in the daily preparation of food. Therefore, educational material with pictures of main herbs with indication on which food they should be added to is a strategy we use to instruct patients for preparing foods with less salt but still very tasty.
- Another practical and very successful experience with patients we carry out occasionally is what we call “cooking hands on session” in which patients cook meals low in salt and explore the taste of herbs to replace the salt.
- Explaining patients how to read and to understand food labels as well as to differentiate sodium from salt are also strategies we use to improve adherence.
- By using test tubes filled with the amount of salt that corresponds to the hidden sodium content in food items such as salami, bologna, ham, cheeses and industrialized broth stock cubes is another approach that enables the patients to be aware on the contribution of these foods on their sodium/salt intake.

#### Advices given to the patients

- When buying salt mark the opening date and the estimated date to finish. For example: for a salt intake equivalent to 4 g/day in a household of 4 people, 1 kg should last 2 months if we consider that the patients make most of their meals at home.
- Salt preference is an acquired taste that can be unlearned. It takes about 6–8 weeks to get used to eating food with much lower quantities of salt, but once it’s done, it is actually difficult to eat foods like potato chips because they taste way too salty.
- Natural spices and herbs improve low salt foods flavor. So, feel free to add garlic, onion, pepper, ginger, nutmeg, coriander, basil, parsley, spring onion, rosemary, sage and so on.
- Use fresh, rather than packaged food. Fresh meats and vegetables contain natural sodium, but the content is still much less than the hidden extra sodium added during processing in products like ham, sausages or canned vegetables. If a food item keeps well in the fridge for days or weeks, that’s a tip off that the sodium content is too high.
- Begin reading food labels as a matter of course. Sodium content is always listed on the label. Sometimes the high sugar content in a product like can mask the high sodium content so it’s important to check every label for sodium content.
- Compare various brands of the same food item until you find the one that has the lowest sodium content, since this will vary from brand to brand.
- Select spices or seasonings that do not list sodium on their labels e.g., choose garlic powder over garlic salt.

### Management of potassium intake

Hyperkalemia is a potential cause of sudden death and dialytic emergency in CKD patients. There are no warning signs and when serum potassium levels approach 5 mg/dl, nutritional counseling to lower dietary potassium is indicated. However, other causes for hyperkalemia should also be investigated and corrected such as metabolic acidosis and constipation together with a review of drug therapies that contribute to this condition such as angiotensin-converting enzyme inhibitors, angiotensin receptor blockers, spironolactone, b-blockers, non-steroidal anti-inflammatory drugs and other drug therapies. Furthermore, tissue destruction (e.g., catabolism) as a result of trauma, insulin deficiency or weight loss releases potassium from intracellular space and results in hyperkalemia in these patients [46].

#### Recommendation of potassium intake

When hyperkalemia cannot be solved by therapy adjustments, a dietary intervention is often necessary. The first step is to know the most important sources of potassium in a typical diet of the region and to investigate the patient usual intake of these foods.

Potassium is widely distributed in foods, but the main sources are those of vegetable origin such as fruits, vegetables, legumes and nuts. As these foods are important sources of fiber, vitamins, minerals and other important nutrients for health in general, a severe restriction is not recommended.

Patients should be informed not only about the foods containing a significant amount of potassium, but also how much they should consume of that. The information can be passed individually during the appointments, as well as in groups using material with pictures dividing the foods according to their amount of potassium. Both strategies work well and one strengthens the other. In general in clinical practice, the initial guidance for NDD-CKD patients is as follows:

- **Fruits:** A list of fruits divided according to a high or low potassium content in a medium portion size is provided to the patient, we classify fruits in two groups (high or low potassium content) according to a medium portion size and recommend patients to choose one portion daily of a fruit high in potassium or two to three portions of fruits with low content of potassium (Table 6).

**Table 6:** Fruits and raw vegetables according to potassium content

Foods low or medium in potassium (<5.0 mEq/portion)	
Fruits	Raw vegetables
1 medium banana “maçã” type^a^	5 lettuce leaves
1 medium persimmon	2 tea saucers of watercress
2 tea saucer of “jabuticaba”^a^	½ small cucumber
1 medium slice of pineapple	1 tea saucer of cabbage
1 orange “lima” type^a^	3 medium radishes
10 strawberries	1 medium red pepper
1 medium apple	1 small tomato
10 “acerolas”^a^	½ medium carrot
½ medium mango	1 tea saucer of raw endive
1 medium pear	
1 medium peach	
1 medium fresh plum	
½ cup of lemon juice	
Foods high in potassium (>5.0 mEq/portion)	
Fruits	Raw vegetables
1 medium banana	1 tea saucer of raw chard
1 medium slice of melon	2 tea saucers of raw cabbage
1 medium orange	3 tablespoons of raw beets
1 medium kiwi	1 tea saucer of chips
½ medium avocado	2 tablespoons of tomato paste
1 mandarin/tangerine	1 tea saucer of fennel
½ cup coconut water	
1 medium slice of papaya	
1 small bunch of grapes	

^a^Typical Brazilian fruits
- **Vegetables:** Two daily portions of those with less potassium or one to two portions daily of the richer in potassium are recommended (Table 6).
- Extraction of potassium from foods: cooking process in water removes about 60 % of the potassium content of vegetables and fruits [47] so we do not restrict them, but advice to peel off and chop vegetables into small pieces and use plenty of water for cooking, which must be discarded after the process.
- **Soups:** Cook the broth in a saucepan and vegetables in another. Once the vegetables are cooked, discard the water and add the vegetables to the broth before serving. Firstly, cook the vegetables and discard the water. Then, add more water to end the cooking process.
- **Fried vegetables:** Cook vegetables in water before frying them such as kale, cabbage, potato, cassava.
- **Other potassium rich foods:**
  Patients should be advised about the high content of potassium of other foods in order to control their intake. Except for the beans, which are daily consumed by Brazilians, the following remaining food items are advised to be avoided for daily use:
  Beans (mainly the Brazilian beans), lentils, chickpeas (since most of the potassium goes to the water, avoid the broth)
  - Espresso coffee, powdered milk, coconut water
  - Nuts and seeds: peanuts, walnuts, chestnuts, hazelnuts, almonds, Brazil nuts, flaxseed
  - Concentrate tomato sauce
  - Chocolate, cakes and biscuits made with chocolate;
  - Brown sugar, molasses
  - Dried fruit, concentrated natural fruit juices, fruit jam.
  - Potato chips;
    **Important:** Patients are advised to not using light or diet salt, since they contain potassium.

These are general guidelines and should therefore be adapted according to the serum potassium concentration and the analysis of the patient’s potassium intake, level of renal function and clinical conditions that may contribute to raise serum levels of potassium.

### Final considerations

This paper reflects a 30-year experience of dietitians working in the NDD-CKD program for the management of patients with CKD. During this period, we have learned that a successful dietary management requires time and effort from patients and dietitians and, most important, should be based on the development of partnership and trust. To this end, it is highly important to explain to the patient the reasons for the dietary changes proposed, to use approaches that enable/facilitate the communication and to respect and understand the patient’s limitations, expectations and desires. Therefore, a team of experienced dietitians who are willing to get involved in such a hard but rewarded work is one of the keys for the success in the dietary management.

## Abbreviations

BMI, body mass index; CKD, chronic kidney disease; eGFR, estimated glomerular filtration rate; GFR, glomerular filtration rate; HBV, high biological value; KDIGO, Kidney Disease Improving Global Outcomes; NDD-CKD, nondialysis-dependent chronic kidney disease; PNA, protein equivalent of nitrogen appearance; RAAS, renin angiotensin aldosterone system; REE, resting energy expenditure; PAL, physical activity level

## References

[1] KDIGO 2012 (2013). Clinical practice guideline for evaluation and management of chronic Kidney disease. Kidney Disease Improving Global Outcomes (KDIGO). Kidney Int.

[2] Levey AS, Schoolwerth AC, Burrows NR, Williams DE, Stith KR, McClellan W (2009). Centers for disease control and prevention expert panel. Comprehensive public health strategies for preventing the development, progression, and complications of CKD: report of an expert panel convened by the centers for disease control and prevention. Am J Kidney Dis.

[3] IBGE. Aquisição domiciliar familiar per capita- Brasil e Grandes Regiões. Rio de Janeiro, Brazil: Ministério do Planejamento, Orçamento e Gestão; 2010.

[4] Martins AP, Levy RB, Claro RM, Moubarac JC, Monteiro CA (2013). Increased contribution of ultra-processed food products in the Brazilian diet (1987–2009). Rev Saude Publica.

[5] Paes-Barreto JG, Silva MI, Qureshi AR, Bregman R, Cervante VF, Carrero JJ, Avesani CM (2013). Can renal nutrition education improve adherence to a low-protein diet in patients with stages 3 to 5 chronic kidney disease?. J Ren Nutr.

[6] Kopple JD, Kopple JD, Massry SG (2004). Nutritional management of nondialyzed patients with chronic renal failure. Nutritional management of nondialyzed patients with chronic renal failure.

[7] Levey AS, Adler S, Caggiula AW, England BK, Greene T, Hunsicker LG, Kusek JW, Rogers NL, Teschan PE (1996). Effects of dietary protein restriction on the progression of advanced renal disease in the modification of diet in renal disease study. Am J Kidney Dis.

[8] Kasiske BL, Lakatua JD, Ma JZ, Louis TA (1998). A meta-analysis of the effects of dietary protein restriction on the rate of decline in renal function. Am J Kidney Dis.

[9] Fouque D, Laville M, Boissel JP (2006). Low protein diets for chronic kidney disease in non diabetic adults. Cochrane Database Syst Rev.

[10] Aparicio M, Bouchet JL, Gin H, Potaux L, Morel D, de Precigout V, Lifermann F, Gonzalez R (1988). Effect of a low-protein diet on urinary albumin excretion in uremic patients. Nephron.

[11] Gansevoort RT, de Zeeuw D, de Jong PE (1995). Additive antiproteinuric effect of ACE inhibition and a low-protein diet in human renal disease. Nephrol Dial Transplant.

[12] National Kidney Foundation. K/DOQI Clinical Practice Guidelines for Nutrition in Chronic renal Failure. Am J Kid Dis. 2000;35(6 Suppl 2):1–140. 10.1053/ajkd.2000.v35.aajkd03517 10895784

[13] Fouque D, Vennegoor M, Wee PT, Wanner C, Basci A, Canaud B, Haage P, Konner K, Kooman J, Martin-Malo A, Pedrini L, Pizzarelli F, Tattersall J, Tordoir J, Vanholder R (2007). EBPG guideline on nutrition. Nephrol Dial Transplant.

[14] Mitch WE, Mitch WE, Klahr S (2002). Dietary requirements for protein and calories in the predialysis patient. Handbook of nutrition and the kidney.

[15] Duenhas M, Gonçalves E, Dias M, Leme G, Laranja S (2013). Reduction of morbidity related to emergency access to dialysis with very low protein diet supplemented with ketoacids (VLPD + KA). Clin Nephrol.

[16] Feiten SF, Draibe SA, Watanabe R, Duenhas MR, Baxmann AC, Nerbass FB, Cuppari L (2005). Short-term effects of a very-low-protein diet supplemented with ketoacids in nondialyzed chronic kidney disease patients. Eur J Clin Nutr.

[17] Utaka S, Avesani CM, Draibe SA, Kamimura MA, Andreoni S, Cuppari L (2005). Inflammation is associated with increased energy expenditure in patients with chronic kidney disease. Am J Clin Nutr.

[18] Avesani CM, Cuppari L, Silva AC, Sigulem DM, Cendoroglo M, Sesso R, Draibe SA (2001). Resting energy expenditure in pre-dialysis diabetic patients. Nephrol Dial Transplant.

[19] Cuppari L, de Carvalho AB, Avesani CM, Kamimura MA, Dos Santos Lobão RR, Draibe SA (2004). Increased resting energy expenditure in hemodialysis patients with severe hyperparathyroidism. J Am Soc Nephrol.

[20] Avesani CM, Draibe SA, Kamimura MA, Dalboni MA, Colugnati FA, Cuppari L (2004). Decreased resting energy expenditure in non-dialysed chronic kidney disease patients. Nephrol Dial Transplant.

[21] O’Sullivan AJ, Lawson JA, Chan M, Kelly JJ (2002). Body composition and energy metabolism in chronic renal insufficiency. Am J Kidney Dis.

[22] Avesani CM, Draibe SA, Kamimura MA, Colugnati FA, Cuppari L (2004). Resting energy expenditure of chronic kidney disease patients: influence of renal function and subclinical inflammation. Am J Kidney Dis.

[23] Avesani CM, Kamimura MA, Draibe SA, Cuppari L (2005). Is energy intake underestimated in nondialyzed chronic kidney disease patients?. J Ren Nutr.

[24] Avesani CM, Kamimura MA, Utaka S, Pecoits-Filho R, Nordfors L, Stenvinkel P, Lindholm B, Draibe SA, Cuppari L (2008). Is UCP2 gene polymorphism associated with decreased resting energy expenditure in nondialyzed chronic kidney disease patients?. J Ren Nutr.

[25] Kamimura MA, Draibe SA, Dalboni MA, Cendoroglo M, Avesani CM, Manfredi SR, Canziani ME, Cuppari L (2007). Serum and cellular interleukin-6 in haemodialysis patients: relationship with energy expenditure. Nephrol Dial Transplant.

[26] Kamimura MA, Draibe SA, Avesani CM, Canziani ME, Colugnati FA, Cuppari L (2007). Resting energy expenditure and its determinants in hemodialysis patients. Eur J Clin Nutr.

[27] Bazanelli AP, Kamimura MA, da Silva CB, Avesani CM, Lopes MG, Manfredi SR, Draibe SA, Cuppari L (2006). Resting energy expenditure in peritoneal dialysis patients. Perit Dial Int.

[28] Bazanelli AP, Kamimura MA, Vasselai P, Draibe SA, Cuppari L (2010). Underreporting of energy intake in peritoneal dialysis patients. J Ren Nutr.

[29] Kamimura MA, Avesani CM, Bazanelli AP, Baria F, Draibe SA, Cuppari L (2011). Are prediction equations reliable for estimating resting energy expenditure in chronic kidney disease patients?. Nephrol Dial Transplant.

[30] Baria F, Kamimura MA, Avesani CM, Lindholm B, Stenvinkel P, Draibe SA, Cuppari L (2011). Activity-related energy expenditure of patients undergoing hemodialysis. J Ren Nutr.

[31] Avesani CM, Trolonge S, Deléaval P, Baria F, Mafra D, Faxén-Irving G, Chauveau P, Teta D, Kamimura MA, Cuppari L, Chan M, Heimbürger O, Fouque D (2012). Physical activity and energy expenditure in haemodialysis patients: an international survey. Nephrol Dial Transplant.

[32] Lambers Heerspink HJ, de Borst MH, Bakker SJ, Navis GJ (2013). Improving the efficacy of RAAS blockade in patients with chronic kidney disease. Nat Rev Nephrol.

[33] de Brito-Ashurst I, Perry L, Sanders TA, Thomas JE, Dobbie H, Varagunam M, Yaqoob MM (2013). The role of salt intake and salt sensitivity in the management of hypertension in South Asian people with chronic kidney disease: a randomised controlled trial. Heart.

[34] McMahon EJ, Bauer JD, Hawley CM, Isbel NM, Stowasser M, Johnson DW, Campbell KL (2013). A randomized trial of dietary sodium restriction in CKD. J Am Soc Nephrol.

[35] Vegter S, Perna A, Postma MJ, Navis G, Remuzzi G, Ruggenenti P (2012). Sodium intake, ACE inhibition, and progression to ESRD. J Am Soc Nephrol.

[36] Lambers Heerspink HJ, Holtkamp FA, Parving HH, Navis GJ, Lewis JB, Ritz E, de Graeff PA, de Zeeuw D (2012). Moderation of dietary sodium potentiates the renal and cardiovascular protective effects of angiotensin receptor blockers. Kidney Int.

[37] McMahon EM, Campbell KL, Mudge DW, Bauer J (2012). Achieving salt restriction in chronic kidney disease. Int J Nephrol.

[38] Jain M, Howe GR, Rohan T (1996). Dietary assessment in epidemiology: comparison of a food frequency and a diet history questionnaire with a 7-day food record. Am J Epidemiol.

[39] Bingham SA (1991). Limitations of the various methods for collecting dietary intake data. Ann Nutr Metab.

[40] Elliot P, Brown I. Sodium Intakes Around the World: Background Document Prepared for the Forum and Technical Meeting on Reducing Salt Intake in Populations. Geneva: World Health Organization; 2006.

[41] Sarno F, Claro RM, Levy RB, Bandoni DH, Monteiro CA (2013). Estimated sodium intake for the Brazilian population, 2008–2009. Rev Saude Publica.

[42] Kidney Disease Outcomes Quality I (2004). K/DOQI clinical practice guidelines on hypertension and antihypertensive agents in chronic kidney disease. Am J Kidney Dis.

[43] Stevens PE, Levin A (2013). Kidney disease: improving global outcomes chronic kidney disease guideline development work group M. Evaluation and management of chronic kidney disease: synopsis of the kidney disease: improving global outcomes 2012 clinical practice guideline. Ann Intern Med.

[44] Levin A, Hemmelgarn B, Culleton B, Tobe S, McFarlane P, Ruzicka M, Burns K, Manns B, White C, Madore F, Moist L, Klarenbach S, Barrett B, Foley R, Jindal K, Senior P, Pannu N, Shurraw S, Akbari A, Cohn A, Reslerova M, Deved V, Mendelssohn D, Nesrallah G, Kappel J, Tonelli M, Canadian Society of Nephrology (2008). Guidelines for the management of chronic kidney disease. CMAJ.

[45] Whelton PK, Appel LJ, Sacco RL, Anderson CA, Antman EM, Campbell N, Dunbar SB, Frohlich ED, Hall JE, Jessup M, Labarthe DR, MacGregor GA, Sacks FM, Stamler J, Vafiadis DK, Van Horn LV (2012). Sodium, blood pressure, and cardiovascular disease: further evidence supporting the American heart association sodium reduction recommendations. Circulation.

[46] Beto JA, Bansal VK (2004). Medical nutrition therapy in chronic kidney failure: integrating clinical practice guidelines. J Am Diet Assoc.

[47] Cuppari L, Amancio OMS, Nobrega M (2004). Preparo de vegetais para utilização em dieta restrita em potássio. Nutrire.

[48] Sargent JA, Gotch FA (1979). Mass balance: a quantitative guide to clinical nutritional therapy. I. The predialysis patient with renal disease. J Am Diet Assoc.

[49] Maroni BJ, Steinman TI, Mitch WE (1985). A method for estimating nitrogen intake of patients with chronic renal failure. Kidney Int.

